# Progress of polymer-based strategies in fungal disease management: Designed for different roles

**DOI:** 10.3389/fcimb.2023.1142029

**Published:** 2023-03-22

**Authors:** Siyu Wu, Wenlai Guo, Bo Li, Huidong Zhou, Hongqi Meng, Junyi Sun, Ruiyan Li, Deming Guo, Xi Zhang, Rui Li, Wenrui Qu

**Affiliations:** ^1^ Department of Hand Surgery, The Second Hospital of Jilin University, Changchun, China; ^2^ Department of Cardiovascular Surgery, The Second Hospital of Jilin University, Changchun, China; ^3^ Changchun American International School, Changchun, China; ^4^ Orthpoeadic Medical Center, The Second Hospital of Jilin University, Changchun, China; ^5^ Jilin Provincial Key Laboratory of Orhtopeadics, Changchun, China; ^6^ Department of Burn Surgery, The First Hospital of Jilin University, Changchun, China

**Keywords:** antimicrobial polymers, drug delivery system, biomaterial, antifungal agents, mycoses

## Abstract

Fungal diseases have posed a great challenge to global health, but have fewer solutions compared to bacterial and viral infections. Development and application of new treatment modalities for fungi are limited by their inherent essential properties as eukaryotes. The microorganism identification and drug sensitivity analyze are limited by their proliferation rates. Moreover, there are currently no vaccines for prevention. Polymer science and related interdisciplinary technologies have revolutionized the field of fungal disease management. To date, numerous advanced polymer-based systems have been developed for management of fungal diseases, including prevention, diagnosis, treatment and monitoring. In this review, we provide an overview of current needs and advances in polymer-based strategies against fungal diseases. We high light various treatment modalities. Delivery systems of antifungal drugs, systems based on polymers’ innate antifungal activities, and photodynamic therapies each follow their own mechanisms and unique design clues. We also discuss various prevention strategies including immunization and antifungal medical devices, and further describe point-of-care testing platforms as futuristic diagnostic and monitoring tools. The broad application of polymer-based strategies for both public and personal health management is prospected and integrated systems have become a promising direction. However, there is a gap between experimental studies and clinical translation. In future, well-designed *in vivo* trials should be conducted to reveal the underlying mechanisms and explore the efficacy as well as biosafety of polymer-based products.

## 1 Introduction

Fungi, organisms that form their own kingdom in the domain of Eukarya, have an estimated 2.2 to 3.8 million species (Sun et al., 2020). Only a small number of genera and species are pathogenic to humans. Some may cause severe diseases and death in hosts with weakened immune systems. Whereas others infest a large population around the world, generally immunocompetent, and cause a benign, topical, self-limiting infection, some of which are global, and others are localized (Ashraf et al., 2020). For example, studies have shown that superficial infections of the skin and nails affect 20–25% of the world’s population, and up to 75% of women have experienced vulvovaginal candidiasis at least once in their lifetime (Sobel et al., 1998; Havlickova et al., 2008). Invasive fungal disease (IFD), a systemic, generalized, deep-seated, visceral, and severe fungal infection, is a global human health challenge (De Pauw et al., 2008). Notably, there is a significant increase in the susceptible immunocompromised population, due to the rise in transplant recipients, cancer patients, and people with chronic diseases such as diabetes (Rayens et al., 2022a). Studies have also reported an increase in fungal infections, such as pulmonary aspergillosis and invasive candidiasis during the coronavirus disease 2019 pandemic, due to an increase in risk factors such as damaged innate defense, use of steroids, and protracted invasive mechanical ventilation (Baddley et al., 2021; Devnath et al., 2021). Considering the associated high risk of mortality, there is an urgent need for development of effective preventive methods, early diagnosis tolls and efficacious treatment modalities (Chiurlo et al., 2021; Mitaka et al., 2021).

To date, however, only a handful of antifungal agents have been identified, owing to the similarity between eukaryotic cells of the host and fungi. Furthermore, some are unavailable for patients with comorbidities due to the risk of severe side effects or drug-drug interactions. Moreover, the frequent and prophylactic use of these drugs has generated an “arms race” of acquired resistance. Although pharmacological research seeking to identify new antifungals with novel modes of action such as glucan synthase inhibitor has shown promise, no clinical translation has been achieved yet (Van Daele et al., 2019; Davis et al., 2020). Interdisciplinary enhancement of conventional drugs with novel materials could be a faster option.

Polymers have been widely used in such applications owing to their highly controlled properties. This has been achieved through choices of various monomers, different chain lengths, and subsequent on-demand functionalization. To date, some natural and synthetic polymers with remarkable biocompatibility and biodegradability, including chitosan and poly(ethylene glycol) (PEG), have been approved for drug excipient application (Alam et al., 2014; D'souza and Shegokar, 2016). Polymer-based novel delivery systems have also shown remarkable efficacy and reduced toxicity. Numerous convenient dosage forms, based on the properties of polymers, can not only enhance topical delivery but also improve patient compliance during the long course of treatment. Moreover, polymeric materials with innate antifungal activities have shown efficacy against multidrug-resistant fungi. Notably, such treatment only represents one passive aspect in management of fungal diseases. Therefore, there is need for development of more proactive methods such as vaccination and self-testing. Furthermore, functionalized polymers have potential as strong adjuvants and key integrators of elements in biosensing devices.

**Scheme 1:**
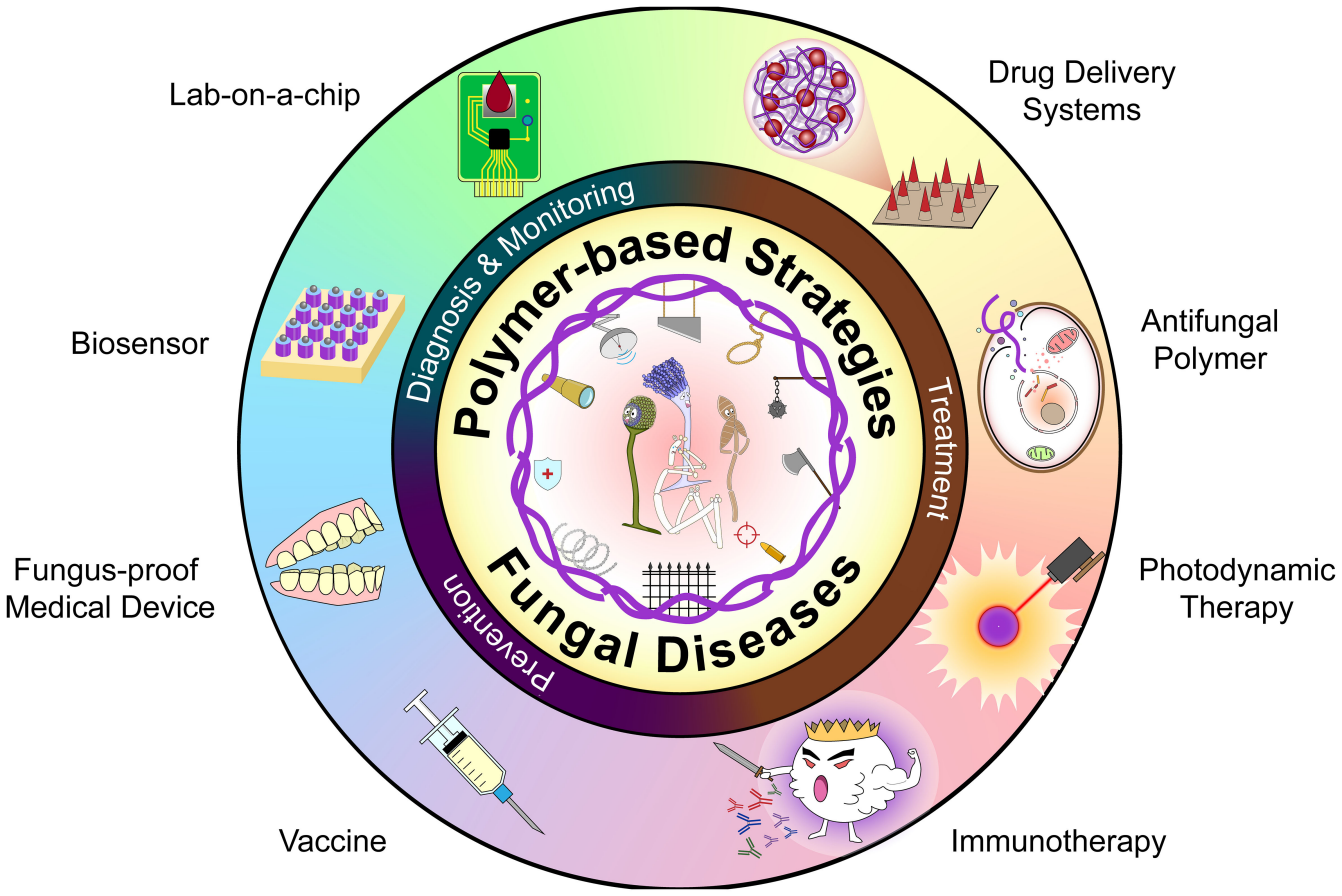


So far, this field has been reviewed with emphasis on nano-scaled composites primarily used as drug delivery systems and synthetic materials with antifungal activity (Du et al., 2021; Nagaraj et al., 2021; Ntow-Boahene et al., 2021). In this review, we describe polymer-based strategies for management of fungal disease, including treatment, diagnosis as well as monitoring and prevention (**Scheme 1**). Firstly, we review common fungal diseases and their conventional treatments, then describe polymer-based drug delivery systems, approaches that depend on the innate antifungal activities of the polymers, and polymer-enhanced photodynamic therapy (PDT). Next, we highlight the progress on active and passive immunization, fungus-proof medical devices, and diagnostic platforms. Finally, we discuss the associated challenges and put forward some recommendations for future directions.

## 2 Human fungal diseases and conventional treatment modalities

### 2.1 Fungal diseases

Fungal diseases are caused by various phylogenetically diverse pathogenic species across phyla within the Kingdom fungi (Jacobsen, 2019). Fungal infections are classified into four clinical types, based on depth of infection, namely superficial, cutaneous, subcutaneous, and systemic infections (Schwartz, 2004). However, this classification only reflects the current state of infection. For example, *Candida albicans* may first colonize the human skin surface or mucosa lining, then invade such sites under certain predisposing conditions, and they tend to infiltrate deeper tissues and disseminate in immunocompromised patients. Therefore, superficial fungal infection may also be a result of invasion and likely to be the initial, local stage of IFD (Hof, 2010).

#### 2.1.1 Superficial fungal diseases

Superficial mycosis, like tinea versicolor and tinea nigra, are infections restricted to the stratum corneum and are associated with little inflammation (Schwartz, 2004). Studies have shown that cutaneous mycosis may affect deeper layers of the epidermis and cutaneous appendages and elicit tissue reaction by the organism or its products (Hsu et al., 2012). Both superficial and cutaneous mycosis are sometimes considered superficial infections and they will be discussed together as superficial fungal diseases in the following parts of this review. Dermatophyte infections are the most common superficial fungal infections in humans (Havlickova et al., 2008). Dermatophytes are filamentous fungi that can utilize keratin as a nutrient source and cause superficial infections in keratinized tissues, including skin, hair, and nails. Their intolerance to body core temperature means that they are almost exclusively localized in the keratinized tissues and seldom cause IFD (Weitzman and Summerbell, 1995; Jacobsen, 2019). Most dermatophytes are from three anamorphic genera, namely *Epidermophyton*, *Microsporum*, and *Trichophyton*. Dermatophytes can also be divided into three ecological groups, based on their natural habitats, namely anthropophilic, zoophilic and geophilic (Weitzman and Summerbell, 1995). Specifically, anthropophilic species colonize human epidermal products and cause mild chronic infections, whereas zoophilic and geophilic species are responsible for acute mycoses with strong inflammatory responses but shorter course of illness (Gnat et al., 2019). Other classification systems, based on phenotypes, genetic relationships, and molecular criteria, are more complicated and less used in the clinical setting (Gnat et al., 2019). Apart from dermatophytes, yeasts and molds have also been shown to cause superficial mycosis, with the elderly reportedly more susceptible to these less common pathogens than the younger population (Wang et al., 2020b). A summary of typical development of skin infections is shown in **Table 1**.

**Table 1:** Typical development of superficial skin infection.

Process	Fungi factors	Host responses
Contact	Passive forces such as hydrophobic and electrostatic interactions	
Adherence	Adhesins on the fungal cell surface Establishment of short and long fibrils (Kaufman et al., 2007) Proteases secretion	Increased cellular turnover
Colonization	Degradation of host tissue structures by hydrolytic enzymes such as keratinolytic proteases and lipases Shaping and adapting to a neutral/alkaline pH (Martinez-Rossi et al., 2017)	Innate immunity Upregulated antimicrobial peptides expression Release of multiple inflammatory cytokines Recruitment of inflammatory cells such as neutrophils, macrophages, and dendritic cells Production and release of melanin granules (Tapia et al., 2014) Activation of sensory neurons (Kashem et al., 2015) Adaptive immunity Activation of immune cells such as Th1 and Th17 (Ma et al., 2021)
Infection	Hyphal extension and invasion Biofilm formation (Burkhart et al., 2002) Binding and activation of host plasmin (Crowe et al., 2003) Induced endocytosis (Waechtler et al., 2012)	

Based on the infection site, superficial fungal diseases can be classified into tinea capitis (scalp), tinea corporis (body), and tinea unguium (nails), among others. Although their clinical presentation varies depending on infection site and pathogenic species, it typically involves a circumscribed scaly and itchy rash (Hsu et al., 2012). Other common signs include changes in appearance of affected nails and hair loss for tinea unguium, and tinea capitis, respectively (Hsu et al., 2012). The mucosa represents another important barrier of our internal milieu. Studies have shown that this area is easily affected under conditions like dampened host immunity or dysbiosis, because fungi, especially *Candida* spp. which form part of the host’s mucosal microbiome, are also opportunistic pathogens (France and Turner, 2017; Witherden et al., 2017; Pellon et al., 2020). Vulvovaginal candidiasis is a common fungal infection that affects up to 75% of women during their lifetime. Notably, recurrent infections reportedly affect 5–8% of women of childbearing age (Sobel et al., 1998; Matheson and Mazza, 2017). The associated symptoms, such as itching and soreness of the vulva, as well as dysuria, dyspareunia, and vaginal discharge, can greatly affect the quality of life (Matheson and Mazza, 2017). Oropharyngeal candidiasis is the most prevalent, recurrent, and indicative opportunistic infection in patients with human immunodeficiency virus (HIV) (Patil et al., 2018). Studies have also implicated a myriad of predisposing factors, local or systemic, such as xerostomia and use of broad-spectrum antimicrobials, in commensal-pathogenic transition (Milsop and Fazel, 2016). The classical presentation of oropharyngeal candidiasis is erasable white plaques on oral mucosa and an erythematous surface left behind. Although, most patients are asymptomatic, some may experience a burning sensation, taste alteration, or bleeding at affected sites (Sharon and Fazel, 2010; Milsop and Fazel, 2016). Some species of *Paracoccidioides*, *Histoplasma*, and *Mucor* are also pathogenic to the mucosa (Marques, 2010). Fungal keratitis is a severe sight-threatening ophthalmic disease that may lead to permanent blindness and eye loss. It often occurs secondary to minor ocular trauma during engagement in agricultural activities, while other reported predisposing factors include previous ocular surgery, ocular surface disease, contact lens use, and common systematic mycosis susceptibility (Brown et al., 2021). Apart from eye inflammation and opacity on the surface of the cornea, patients usually experience pain, discharge, photophobia, and reduced vision (Ansari et al., 2013). Other signs that differentiate this disease from bacterial keratitis include satellite lesions and irregular borders of the ulcer. However, the distinguishing accuracy is only 60–70% even for the experts (Dalmon et al., 2012).

#### 2.1.2 Deep fungal diseases

Primary subcutaneous mycoses are usually induced by traumatic implantation or wound contamination, and may spread to internal organs through lymphatic vessels and the blood stream (Fernandez-Flores et al., 2014; Shields et al., 2019). Studies have shown that patients may be infected after inhaling conidia or mycelial fragments, as well as colonization by opportunistic pathogens like *C. albicans* (Fernandez-Flores et al., 2014; Shields et al., 2019). Other classifications have separated systemic and opportunistic diseases by fungal virulence and host immunity. In some cases, only dimorphic fungal infections are classified as systemic mycoses (Arenas et al., 2012). Both subcutaneous and systemic fungal infections mainly occur in tropical climates and in immunosuppressed patients, due to the fact that invading pathogens are not only restricted but also eliminated in most immunocompetent people unless the inoculum is abundant (Arenas et al., 2012). However, these pathogens reportedly cause more serious clinical problems in immunocompromised individuals, to whom opportunistic fungi become pathogenic and pathogenic ones more virulent. Host factors are significant in probable and possible diagnosis of IFD, if the mycologic evidence are insufficient. They are used to identify patients very prone to fungal infections, that mainly are causes of severe immunosuppression such as the recent history of neutropenia, receipt of transplantation and prolonged use of corticosteroids (Donnelly et al., 2020). Risk factors are about underlying diseases that statistically increase the chance and severity of IFD. In recent years, risk conditions have moved away from HIV infections to people with certain conditions such as diabetes mellitus, respiratory disorders (chronic obstructive pulmonary disease and asthma), and cancer (Rayens et al., 2022a). Some bacterial or viral infections (sepsis, pneumonia, and influenza, among others) as well as organ dysfunction have also been reported (Rayens et al., 2022b). Studies have shown that fungal infections not only doubled the average cost and length of hospital stays in at-risk patients, but also significantly exacerbated severity of the underlying disease and risk of death (Rayens and Norris, 2022).

The role of certain genes and pathways in susceptibility to fungal infections has become a new research hotspot. Notably, polymorphisms in soluble and membrane-bound pattern recognition receptors represent significant innate immune response elements that could affect an individual’s susceptibility to fungal diseases. Previous studies have associated variations among related genes, such as mannose-binding lectin and toll-like receptors, with increased risk of certain fungal infections (Carvalho et al., 2010). Moreover, mutations in genes encoding members of the interleukin family and metabolism of immune cells have also been implicated in susceptibility (Naik et al., 2021)to fungal infections. Therefore, detection and functional characterization of such biomarkers may provide valuable approaches for future development of personalized prevention and treatment strategies for fungal diseases.

Pathogenic fungi invade different sites and cause different clinical presentations. Among them, cutaneous and subcutaneous tissues are commonly involved sites in systemic fungal diseases of some species, and may display different clinical manifestations from primary infections (Fernandez-Flores et al., 2014). The clinical manifestation of IFD could be very insidious or nonspecific, and lead to delayed or missed diagnosis (Danion et al., 2019). Generally, the diagnosis is based on multiple evidences besides host factors, including clinical, radiological, histological and microbiological findings from biopsy and culture (De Pauw et al., 2008; Pappas et al., 2018; Berger et al., 2019). Polymerase chain reaction (PCR) and antigen biomarkers such as galactomannan and β-D-glucan are useful non-invasive methods for microbiological analisis (Badiee et al., 2016). However, these approaches are not readily available in some less developed countries and regions (Falci and Pasqualotto, 2019). To effectively manage global health challenges brought about by fungal diseases, there is a need to urgently develop convenient and low-cost diagnostic tools.

### 2.2 Conventional treatment modalities for fungal diseases

Although pathogenic fungi can be classified into numerous genera with various morphologies, they share similar cell wall and membrane components that distinguish them from mammalian cells. Researchers have exploited these as therapeutic targets, although fungi’s susceptibility to these targets varies between strains (Du et al., 2021). At present, four main classes of antifungal agents namely polyenes, azoles, echinocandins, and pyrimidine analogs, are applied for treatment of systemic mycoses. Agents from other classes, including mitotic inhibitors (griseofulvin), allylamines (terbinafine), aminoacyl transfer RNA synthetase inhibitors (tavaborole), and hydroxypyridone derivatives (ciclopirox), have been employed as alternative drugs for treatment of superficial infections. Details on their underlying mechanisms of action and Food and Drug Administration (FDA) approved dosage forms of representative drugs are listed in **Table 2**.

**Table 2:** Representative antifungal drugs approved by FDA. Rx: prescription drug; OTC: over-the-counter drug.

Categories	Antifungal Drugs	Mechanism of Action	Dosage Forms	Administration Routes	Need of prescription
Polyene	Amphotericin B	Directly binds to ergosterol in the cell membrane of susceptible fungi, forms transmembrane channels, and alters the cell permeability that result in cytoplasm leakage.	Lyophilized powder with sodium desoxycholate for injection	Intravenous	Rx only
			Lyophilized lipid-complex for injection		
			Lyophilized liposomal for injection		
	Nystatin		Tablet	Oral	Rx only
			Suspension		
			Powder	Topical	
			Cream		
			Ointment		
			Suppository	Vaginal	
			Tablet		
	Natamycin		Suspension	Ophthalmic	Rx only
Azoles	Fluconazole	Selectively inhibit the fungal cytochrome P450-dependent enzyme, lanosterol 14-α-demethylase, which convert lanosterol to ergosterol.	Injection	Intravenous	Rx only
			Tablet	Oral	
			Powder for suspension		
	Itraconazole		Capsule	Oral	Rx only
			Solution		
	Clotrimazole		Lozenge	Buccal	Rx only
			Solution	Topical	OTC
			Lotion		
			Cream	Topical; Vaginal	
	Miconazole		Tablet	Buccal	Rx only
			Ointment	Topical	Rx only
			Cream	Vaginal	OTC
			Suppository		
			Insert		
Echinocandins	Caspofungin	Noncompetitively inhibits the β-1,3-D-glucan synthase, resulted in compromised synthesis of glucan and instability of fungal cell wall.	Lyophilized powder for injection	Intravenous	Rx only
	Micafungin		Lyophilized powder for injection	Intravenous	Rx only
Pyrimidine analogs	Flucytosine	After taken up by the fungi, it is converted to fluorouracil, and the active metabolites inhibit cellular metabolism *via* incorporation into RNA or inhibition of thymidylate synthetase.	Capsule	Oral	Rx only
Mitotic inhibitor	Griseofulvin	Inhibits the function of mitotic spindle microtubule.	Tablet	Oral	Rx only
			Suspension		
Allylamines	Terbinafine	Inhibits the biosynthesis of ergosterol on squalene epoxidase enzyme. The accumulation of high concentrations of squalene results in increased membrane permeability.	Tablet	Oral	Rx only
			Granule		
			Cream	Topical	OTC
			Gel		
			Spray solution		
tRNA synthetase inhibitor	Tavaborole	Inhibits protein synthesis by inhibition of leucyl-tRNA synthetase.	Solution	Topical	Rx only
Hydroxypyridone	Ciclopirox	Chelates polyvalent cations such as Fe^3+^ and Al^3+^, resulting in the inhibition of some metal-dependent enzymes related to antioxidant defense.	Gel	Topical	Rx only
			Cream		
			Suspension		
			Shampoo		
			Nail lacquer		

Topical treatment is preferred to oral applications for many superficial infections, due to the fact that the drug bypasses the first-pass elimination, and has also been found to result in lower systemic side effects, and concentrates at the target site (Rotta et al., 2013). Besides, improved personal hygiene and dry, loose-fitting clothing have been shown to accelerate the recovery process (Jartarkar et al., 2022). Surgery is also effective for management of some subcutaneous mycoses, as excision is the most direct method for reducing pathogen load and the only independent predictor of survival (Fernandez-Flores et al., 2014; Mauch et al., 2020). Corneal scraping and penetrating keratoplasty are the most common surgical interventions for refractory or severe fungal keratitis (Ansari et al., 2013). However, systemic treatment is still required for more serious and relapsing local infections for better clinical efficacy (Jartarkar et al., 2022). Although pathogen-targeting antifungal agents remain the first choice for fungal infection management, clinicians have also developed immunomodulating therapies (Ademe, 2020). Repurposing of approved drugs, such as statins and mebendazole, is also under investigation (Joffe et al., 2017; Tavakkoli et al., 2020).

## 3 Polymer-based strategies in fungal disease treatment

### 3.1 Delivery systems for antifungal drugs

Optimization of known drugs coupled with constant development of new ones represents endless pursuits in medical and pharmaceutical research. Considering that many conventional antifungal drugs are hydrophobic, researchers have hypothesized that increasing their aqueous solubility could promote their efficacy. Although formation of conjugates with hydrophilic macromolecules is straightforward, care should be taken not to compromise drug activity (Ravichandran and Jayakrishnan, 2018). Generally, polymer-based carriers are combined with drugs in non-covalent ways, and could be functionalized for controlled tissue distribution, different pharmacokinetics, and reduced toxicity, among others. Optimization of drug dosages, during topical application, represents high potential for improved efficacy and convenient application of these drugs.

#### 3.1.1 Micro and nano-carriers in systemic application

**Figure 1:**
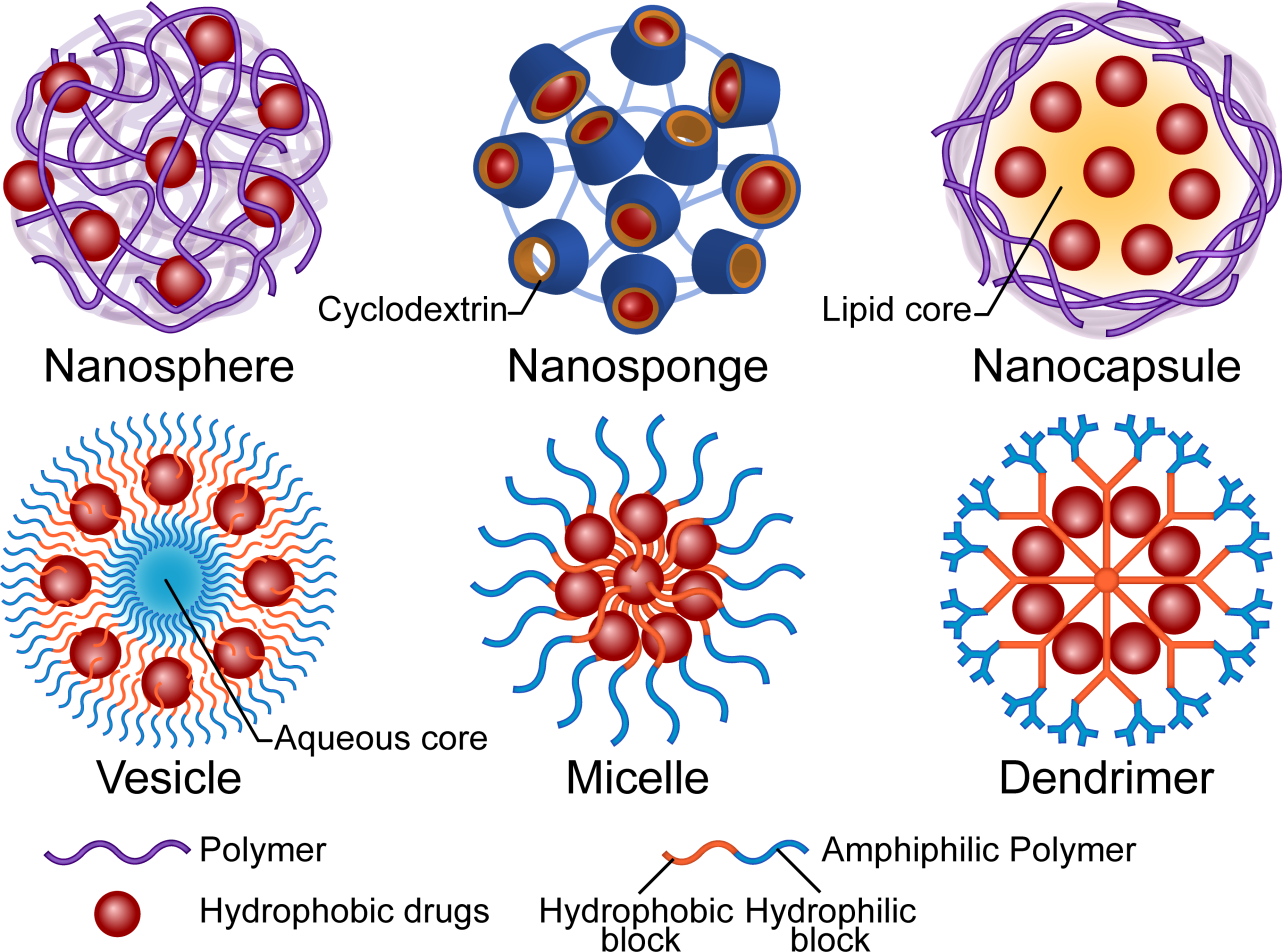
Illustration of the structures and main composition of polymer-based drug carriers. The micro or nano carriers are primarily designed to increase the water solubility, bioavailability and safety of the antifungal drugs. The polymers could be functionalized for controlled drug distribution, pharmacokinetics and new administration route.

Micro and nano-carriers greatly improve the delivery of antifungal drugs, owing to the associated advantages including high dispersibility, protection from premature degradation, and ability to maintain the free drugs at therapeutic levels which lowers toxicity without affecting efficacy. Correct functions, such as improved surface contacting, controlled release, targeted delivery, and improved sensitivity, rely on properties of the material or surface modification. Phospholipids, nonionic surfactants, and polymers are the main organic materials used in drug encapsulation. Among them, lipid-based materials are the most abundant in pharmaceutical research and clinical use (Bulbake et al., 2017; Nene et al., 2021). For example, AmBisome^®^, a unilamellar bilayer liposomal Amphotericin B (AmB) delivery system, has been used as an updated substitute for AmB deoxycholate for over two decades. Although liposomal formulations have advantages, such as low nephrotoxicity and prolonged tissue residence, their low urinary clearance constrains the application for treatment of lower urinary tract infections (Stone et al., 2016). Concerns regarding membrane fusion-induced toxicity and the associated instability have also restricted the clinical translation of other lipid-based formulations (Yu et al., 2017; Nakhaei et al., 2021). Studies have shown that some inorganic carriers, such as metallic nanoparticles (NPs) and carbon nano-tubes, may accumulate in solid organs like the liver and induce repetitive injuries that eventually overwhelm the regenerative capacity and cause irreversible damage (Li et al., 2022). Conversely, polymers are promising materials for carrier construction owing to their excellent biocompatibility and biodegradability. Higher expectations are attached to nano-scaled structures due to their small size and high surface-to-volume ratio, which may enhance their interaction with pathogens (Kamel, 2019). The structures of carriers vary with regards to materials and processes that represent different strategies of encapsulation. A summary of available carrier structures is provided in **Figure 1**.

Polymeric nanospheres consist of a continuous matrix of polymers in which the hydrophobic drugs disperse. Notably, drug release relies on both the diffusion mechanism and the degradation of the polymer matrix. Poly(lactic-co-glycolic acid) (PLGA), in which the hydrolyzed monomers, lactic acid and glycolic acid, are endogenous and easily metabolized (Danhier et al., 2012), represents one of the most favorable and authority-approved polymers for drug delivery. A poriferous structure, namely nanosponge, is a special kind of nanosphere. The complexation property of the three-dimensional structure influences the attraction and release of the molecules (Deng et al., 2021). Cyclodextrins, cyclic oligosaccharides with a hydrophilic outer surface and a hydrophobic inner cavity, are widely used to increase solubility of hydrophobic drugs in water (Varan et al., 2017). Hydroxypropyl-β-cyclodextrin (HP-β-CD) is used in SPORANOX^®^, an itraconazole oral solution, as a molecular inclusion complex. Osmani et al. (2016) solubilized clotrimazole by fabricating the HP-β-CD nanosponge with dimethylcarbonate as a cross-linking agent and found that a higher degree of cross-linking improved the drug entrapment capacity. In the case of nanocapsules, the hydrophobic drug is sealed in the solvent (lipid) core by a biodegradable polymeric membrane, although in a broad sense, nanospheres with a polymeric shell are considered nanocapsules (Mora-Huertas et al., 2010). Studies have shown that apart from properties of the polymer, manufactory methods, choices of the core oil, and components of the dispersion system also influence the parameters of the particle and result in diverse release profiles or permeation of biological barriers (Fiel et al., 2011; Ribeiro et al., 2016; Englert et al., 2020). An initial burst release is commonly observed in the aforementioned formulations, due to the possibility of the drugs to be adsorbed onto the surface of structures. This may be advantageous since it could accelerate reaching the therapeutic concentration, especially for the long-term-release formulations. For sensitive species, NPs’ minimum inhibitory concentration (MIC) may be higher than that of free drugs because of the encapsulation (Moraes Moreira Carraro et al., 2017). However, for resistant fungi, the nanostructured carriers may reverse the resistance *via* multiple mechanisms, such as surface interactions, thus prevent recognition by efflux pumps (Bianchin et al., 2019). A previous study found that antifungal activity was enhanced by both size-dependent NP internalization in whole and the nearby release (Patel et al., 2011). Notably, the authors demonstrated that 200nm-sized itraconazole-loaded NPs were not only more efficiently taken up by *Aspergillus flavus* compared to 1200nm-sized ones, but also displayed better antifungal activity at low concentrations (Patel et al., 2011). Nevertheless, more research is needed on drugs with different located targets and mechanisms of action.

Unlike the entanglement of polymer chains in the aforementioned NPs, amphiphilic polymers may also encapsulate drugs in a relatively regular arrangement. Polymeric vesicles, also known as polymersomes, not only have the most liposome-like structure (comprising a self-assembling amphiphilic block copolymer membrane and an aqueous core) but are also available for encapsulation of both hydrophilic and hydrophobic molecules. The complex and entangled polymeric membrane confers polymeric vesicles with high stability and a tunable permeability (Zhang and Zhang, 2017). Polymeric micelles, which are also based on amphiphilic polymers, are self-assembled. In contrast to vesicles, the core of micelles is the hydrophobic tails of the polymers and the hydrophilic heads unite as a shell. Their stability depends on entropy gain on micellization. Small hydrophobic drugs can be physically entrapped in the core based on the “like dissolves like” principle or conjugated to the polymer tail (Hwang et al., 2020). The micelles tend to dissociate and release the drugs when concentration of the amphiphilic macromolecules decreases. Studies have shown that an increase in the assembly’s inherent hydrophilicity may push the cargo out of the core due to hydrolysis or other chemical reactions (Guo et al., 2020). Researchers have used dendrimers, a class of hyperbranched polymers, to construct unimolecular micelles that are resistant to dilution-induced dissociation. The core of the dendrimer may be modified with hydrophobic blocks to increase the loading of hydrophobic drugs, while the hydrophilic branches may also be functionalized for long-circulating or targeted delivery (Hwang et al., 2020).

Previous studies have also shown that polymer-based surface modification of lipid, polymeric or inorganic carriers can improve drug delivery capabilities. For example, one study revealed that poly(dopamine) coating could facilitate intradermal delivery of terbinafine-loaded nanostructured lipid carriers *via* the follicular route (Chen et al., 2020). Another demonstrated that chitosan coating not only resulted in prolonged ocular retention but also penetration of AmB-loaded nanostructured lipid carriers (Fu et al., 2017). As for systematic application, particularly controlled-release formulations, researchers have attempted to prevent being phagocytized by the mononuclear phagocyte system. PEG is one of the most widely studied materials for surface modification, especially for delivery of anticancer agents. PEGylation forms a hydration layer around the NPs that reduces the interaction with plasma proteins and recognition by phagocytes, thereby causing prolonged stealthy circulation and stable stereo structure (Gagliardi et al., 2021). However, a recent study described the side effects associated with PEG use, such as induction of PEG antibodies. Nevertheless, development of better alternatives has achieved some progress (Simon et al., 2020).

Active targeting, a concept derived from anticancer agent delivery, has been suggested as an effective approach for reducing effective dose and toxicity by the improved local concentrations of antifungal drugs in close proximity to fungi. Affinity ligands not only direct the binding but may also improve cellular uptake of nano-carriers. In one study, CKR12, a mutant of the antibacterial core peptide, was hybridized with PLGA to an amphiphilic block copolymer. Results showed that it successfully formed a micelle structure for miconazole encapsulation, and reduced the MIC to one-eighth compared to free drug (Mori et al., 2021). The carrier itself showed a MIC of 0.24 µmol/L, indicating that it synergistically interacted with the antifungal drug (Mori et al., 2021). Mammalian immune receptors, Dectin-1 and Dectin-2, have also been used to direct the carriers to the glucans and mannans in fungal cell walls (Ambati et al., 2019a; Ambati et al., 2019b). Apart from increasing dissolution in luminal fluids, carriers can be applied to improve oral absorption of hydrophobic drugs *via* different mechanisms. Quaternary ammonium palmitoyl glycol chitosan, a mucoadhesive amphiphilic polymer, was developed for oral griseofulvin and AmB delivery. The polymer formed micelles when loaded with griseofulvin and increased the maximum plasma concentration by 480% (Siew et al., 2012). The presumable mechanism was that the bioadhesive micelles were confined to the absorptive regions of the upper gastrointestinal tract, leaving a longer absorptive time window before being washed away by chyme. The tight junctions were not opened, but transcellular transport was promoted (Siew et al., 2012). Combination with AmB resulted in formation of polyelectrolyte complexes between the polymer’s positively charged quaternary ammonium groups and AmB’s carboxylic groups. Adding on the hydrophobic interactions between the palmitoyl chains, this NP formulation showed exceptional stability. The plasma level of oral NPs was 2-fold higher than that of liposome formulation, and higher portions of the drug were delivered to the spleen and lungs. Although oral NPs resulted in lower AmB levels compared to intravenous liposomes in tested organs, they displayed similar efficacy in treatment of visceral leishmaniasis, aspergillosis, and systemic candidiasis in animal models (Serrano et al., 2015). This formulation also exhibited increased anti-biofilm efficiency for better penetration (Alakkad et al., 2022). Despite the massive designs in drug delivery systems, only a few studies have presented results of *in vivo* testing. It is possible that the drugs’ releasing curve could be very different from *ex vivo* testing due to the presence of enzymes and phagocytes. Besides, the distribution of carriers may be significantly affected by natural barriers such as the blood-brain and blood-ocular barriers (Nowak et al., 2020; Swetledge et al., 2021). Therefore, further elucidation of the distribution and serum concentrations *in vivo* is imperative to comprehensive evaluation of antifungal delivery systems.

#### 3.1.2 Advanced dosage forms for topical application

Considering that most fungal colonization and infection are topical and superficial, clinicians prefer topical application of antifungals for increased concentration at affected sites and reduced systematic toxicity. Solutions, creams, ointments, and gels are conventional forms for topical application. Polymers such as PEG and carbomers are widely used in such formulations as auxiliary substances for stabilizing, thickening, emulsification, gel-forming, etc. Novel dosage forms are in development for more convenient use, patients’ experience, and improved efficacy. The roles of the polymers are also extended beyond excipients to vehicles and even part of a compound prescription.

##### 3.1.2.1 Hydrogels and films

Hydrogels, systems composed of a three-dimensional cross-linked network of hydrophilic polymers with the space between the chains filled with water (Ahmed, 2015), are semisolids with very tunable rheological characterizations for topical spread. On the other hand, films are two-dimensional dry versions of polymer networks, characterized by interpenetration and coalescence during the solvent casting process (Felton, 2013). Both systems can easily encapsulate drugs or carriers in their polymer matrix or through chemical bonds for controlled topical release.

The water in hydrogels may hydrate the stratum corneum of skin, thereby causing reversible degradation of the barrier function and modulating drug permeation (Charalambopoulou et al., 2004; Roberts et al., 2021). Formulations can also be dehydrated for better storage, and rehydrated with water, buffer, or body fluid like saliva. They may also provide evaporative cooling to the irritated area thereby relieve the discomfort. Since long-time occlusion and moisture violate the main demands of tinea treatment, namely clean and dry, highly crosslinked hydrogel patches are less recommended for skin application. Besides, such dosage forms are not suitable for large-area use. Therefore, the formulations for dermatophytosis are usually developed into viscoelastic liquids that dry and form a film on the skin for sustained drug release. Studies have shown that permeation and delivery profiles may be optimized during the period of drug supersaturation (Frederiksen et al., 2016). Notably, nanogels have been shown to outperform commercial creams in dermal retention due to a combination of the advantages of stratum corneum hydration and the effects of NPs (Ul Hassan et al., 2022).

Chemical groups on the polymer chain may provide extra adhesion on the body surface *via* non-covalent bonds, such as hydrogen bonds and cation-π interactions (Zhang et al., 2022a). Natural and modified mucoadhesive polymers, based on covalent bonds with mucosa composition, are also highly developed for drug delivery and surgical use (Brannigan and Khutoryanskiy, 2019). The adhesion is largely dependent on their swelling capacity for the relaxation of the network and exposure of such chemical groups. Szekalska et al. (2021) encapsulated posaconazole in fucoidan-gelatin microparticles by spray drying. When moisturized with body fluid simulates, the formulation with more carboxyl groups and a higher swelling ratio exhibited higher detachment force and work adhesion, indicating that hydrogen bonds have a significant contribution. In another study, Ozkahraman et al. (2022) modified polysaccharides with thiolated agents, L-cysteine and 3-mercaptopropionic acid, to generate disulfide bonds between thiol groups on the polymer chains and cysteine residues on glycoproteins in the mucosa, and found that this markedly improved the mucoadhesive properties. Further, they fabricated a drug-loading buccal film patch and found that thiolated group amounts were correlated with the force required to detach the patches from the mucosal membrane.

The bond-induced retention of polymer chains is highly compatible with ocular drug delivery to overcome physiological specificities such as tear secretion and nasolacrimal drainage (Patel et al., 2013). The pseudoplastic behavior of such hydrogels minimizes discomfort, whereas viscosity decreases during the high shear rate of eye blinking (Ahmed and Aljaeid, 2017). Suspension of mucoadhesive polymers can also mediate sustained drug release. A double-conjugated polymer was synthesized with chitosan, cyclodextrin, and a catechol for econazole delivery. Corneal penetration enhancement of the drug was induced by opening of corneal epithelial tight junctions as well as prolonging residence time, and a twice-daily dosage was enough to generate satisfactory therapeutic efficacy in fungal keratitis model (Shi et al., 2022b).

*In situ* gelation has been proposed for easy administration. The sol-gel transition has to be induced by conditions and components of the site such as temperature, pH, electrolytes, or enzymes. Ion-sensitive formulations are suitable for ocular delivery due to presence of mono and divalent cations such as Na^+^, K^+^, Mg^2+^, and Ca^2+^ in tear. A previous study showed that an ion-sensitive formulation of gellan gum and κ-carrageenan outperformed the commercially available solution in voriconazole pharmacokinetics with regards to retention on both ocular surface and other internal structures (Diaz-Tome et al., 2021).

Thermosensitive formulations with low viscosity at room-temperature for high spreadability and gel at body temperature have shown promise in vaginal delivery. Poloxamers, a series of highly commercialized amphiphilic block copolymers, have a concentration-dependent gelation temperature and the extended retention time is attributed to bounds between hydrophilic oxide groups and oligosaccharide chains of the mucosal membrane (Osmani et al., 2016). Studies have suggested that reversible gels may transform back to sol, resulting from dilution by vaginal fluids, and leak (Yu et al., 2011). However, polymeric films may overcome such disadvantages and provide an accurate dose (Cervi et al., 2022). Ocular delivery systems do not have such concerns, although the films’ dosage form may be excessively irritating. Researchers developed a thermosensitive hydrogel, based on the copolymer of poly(N-isopropylacrylamide) and hyaluronic acid (HA), for ketoconazole delivery. The formulation had a gelation temperature of 33 °C, and it showed no signs of irritation (Zhu et al., 2018).

Functionalized hydrogels may also interact with physical enhancements such as heat, magnetic or electric fields (Lee et al., 2018; Ribeiro et al., 2021). Iontophoresis is a non-invasive method in which charged drugs are repelled and driven into the body for topical or systemic application in a given electric field (Dhote et al., 2012; Karpinski, 2018). Optimal molecules for iontophoresis should be small and hydrophilic, properties that most antifungal agents do not have (Dhote et al., 2012). An et al. (2020) encapsulated fluconazole in surfactant-based nanocarriers together with oleylamine for enhanced charge. They loaded the product into an electroconductive poly(vinyl alcohol) (PVA) -poly(pyrrole) hydrogel as a drug reservoir and electrode patch, then introduced the reverse electrodialysis technology into the system as a battery for portable and disposable use.

##### 3.1.2.2 Nail lacquers

Nail lacquers are widely applied as cosmetics and are composed of a polymeric film former, solvents, and other chemical compounds for better attachment, plasticity, color, etc. After application, the solvent volatilizes, leaving a thin film on the nail plate for decoration and protection. Nail lacquers with onychomycosis have been long studied and approved for commercialization. Loceryl^®^ and Penlac^®^ contain 5% amorolfine and 8% ciclopirox, respectively, and have been in use since the 1990s (Shanbhag and Jani, 2017). Lacquers form thin films with concentrated drugs fixed on the nail surface, providing a relatively sustained release. They stick more strongly compared with gels, creams, and paste. In addition, potential advantages include occlusion and reinfection prevention (Kataria et al., 2016). However, the repetitive application and complex removal procedures, which take tens of months, could compromise patient compliance (Aggarwal et al., 2020b). Two main directions of formula optimization are: easier removal with accelerated drug delivery, or reducing reapplication frequency by improved drug load and attachment.

The nail plate is a cornified epithelial structure that consists of various types of keratins with a total lipid content of 0.1~1% (De Berker et al., 2000; Walters et al., 2012). Thus, it is also described as a hydrogel with an additional lipophilic pathway that acts as a reservoir under certain conditions (Laube et al., 2019). Therefore, efficient drug transfer from the film to the nail plate is a strategy for sustained release that fully uses the nail’s innate proprieties.

Since fungi infect the nail plate, bed, and surrounding soft tissue, trasungual drug delivery and intralingual drug retention are needed (Zaias and Rebell, 2004). Several modifications of delivery agents, physical and chemical, have been studied and reviewed in previous works (Murdan, 2008; Angelo et al., 2017; Aslam et al., 2021). One key finding is that hydration causes the keratin network to swell and expand, leading to larger pores allowing small polar molecules (Chouhan and Saini, 2012)to permeate. Water is therefore recognized as an effective permeation enhancer that does not break bonds and increases the flexibility of nails (Walters et al., 2012; Saner et al., 2014). Chemical enhancers that disrupt keratin disulfide linkages are designed to increase nail hydration and pore sizes (Hafeez et al., 2013). Chouhan and Saini formulated a cellulose-based terbinafine lacquer and observed that with the addition of HP-β-CD, drug permeation flux was increased, owning to hydration of nails and improved solubility of the hydrophobic drug. Thus, the oligomer is considered a suitable and safe nail improvement strategy for antifungal transungunal delivery (Chouhan and Saini, 2014). HP-β-CD complexation was used to improve the physical stability of thymol, a highly volatile antifungal molecule, and increase its retention in the film (Cunha-Filho et al., 2021). Similar lacquers were developed in sulfur, silicon, and biotin supplements to treat nail inflammatory disorders (Fernandez-Campos et al., 2020). The release of drugs is affected by the characteristics and concentration of the film former, plasticizer, solvent, and drug (Sveikauskaite and Briedis, 2017; Cutrin-Gomez et al., 2018; Valdes et al., 2018; Aggarwal et al., 2020a). Interactions between drug and film-forming systems reduce the release or inactivation and hence should be avoided in quick delivery systems (Sveikauskaite and Briedis, 2017). As for easier removal of the film, there is no substitute for washable formulations for easier film removal. Eudragit E, an acidic-water-soluble acrylic resin, was used to form a film with citric acid and glycerin as plasticizers. The presence of citric acid reduces local pH and increases the aqueous solubility of the polymeric matrix (Cunha-Filho et al., 2021).The stability of the formula at room temperature is a critical factor for easier application because patients may skip treatment if the lacquer has to be refrigerated.

The alternative direction is to minimize the frequency of reapplication. The film must be durable, waterproof, and firmly adhered to the nail. Most importantly, sufficient drugs are needed to sustain long-term release and to maintain an effective concentration.

Interactions between the nail plate and film-forming system were designed to increase adhesion. Valdes et al. (2017) were the first to apply poly(urethane) in therapeutic nail lacquers for terbinafine delivery. The increase in adhesion was attributed to the high isosorbide proportion in the polymer chain, which forms hydrogen bonds with keratin. The selected formulation exhibited a degree of flaking in the lattice pattern of 1.8%, and only small flakes detached in the cross-cut area. A similar poly(urethane) formula loaded with ciclopirox had even higher adhesion. This was attributed to ethyl acetate as a solvent that increased the formation of hydrogen bonds. The experimental formulations performed well compared to commercial lacquer in terms of drug deposit, permeation, and antifungal trials (Valdes et al., 2018). A bilayer formulation comprises a drug-loading base layer with a hydrophilic film former such as hydroxypropyl methylcellulose and water-resistant drug-free coat (Hasan et al., 2018; Rahman et al., 2021). The hydration and swelling of polymeric films increased adhesion, whereas the hydrophobic layer increased durability (Rahman et al., 2021).

Kerai et al. (2015); Kerai et al. (2016) explored the use of ultraviolet (UV)-curable gels, technique used in nail cosmetic industry for pharmaceutical applications. The formulation contains acrylate-and methacrylate-based monomers, polymerization photo-initiating system, a solvent, and an antifungal agent such as amorolfine or terbinafine hydrochloride. The UV-cured films showed promising results *in vivo*. Herein, experimental films lasted for two weeks, whereas commercial lacquers lasted for three days. Moreover, they also outperformed the commercial type in terms of occlusion by reducing trans-onychia water loss by approximately 20%, reducing the lag time, and increasing permeability. Chemical permeation enhancers were also introduced into the system, and they influenced both the vehicle and the nail plate. Notably, the extent of polymerization had no effect on drug release and ungual permeation (Kerai et al., 2018). Although previous findings reported that UV have inhibitory effect on *Trichophyton rubrum*, the wavelength segment used for gel curing showed no inhibition (Cronin et al., 2014; Kerai et al., 2015).

Generally, hydrophilic films increase drug diffusion through swelling and relaxation of the polymer network (Thapa et al., 2016). Conversely, highly cross-linked networks and polymer concentrations in the film result in thicker and denser films that slow down drug release (Valdes et al., 2018). The drug retention within the nail plate function as a pool where most of the antifungal agents are stored. However, the permeation of the nail plate regulates the in and out flow of the pool. Higher permeability shortens the lag time and depletes the drug reservoir rapidly (Khattab and Shalaby, 2018). A combination of hydrophobic and hydrophilic polymers in the formulation balances the drug retention and permeability across the nail plate (Thatai and Sapra, 2018).

##### 3.1.2.3 Microneedle patches

Compared with the surface application, microneedles (MNs) are a more promising drug delivery system because of their reliable mechanical penetration of stratum corneum (Bubic Pajic et al., 2021). Besides, the dose form of patches provides more accurate control of active ingredient content compared with other topical formulations such as creams and gels, but they are not applicable for large-area use. Biodegradable polymer MNs are preferred for their biocompatibility, low cost, and lack of sharp hazardous waste (Larraneta et al., 2016). Although it is generally regarded as a transdermal system for systemic delivery, as the drugs can enter the circulation through blood vessels in the upper papillary dermis, the lipophilicity of most antifungal drugs tends to locate them in the lipid intercellular regions of epidermal keratinocytes and stratum corneum (Dhote et al., 2012; Larraneta et al., 2016). Peng et al. (2021b) fabricated a dissolving MN patch with poly(vinylpyrrolidone) (PVP) and PVA for micronized AmB particle delivery. An *in vivo* study on mice have shown that of MN application, the maximum drug level in the skin was achieved 20 hours earlier and 410-fold higher compared with the intravenous route. The drug level remained 22-fold higher than the peak of intravenous injection on the seventh day. The biodistribution and pharmacokinetic study showed little systemic exposure in kidney, liver, spleen, and plasma.

**Figure 2:**
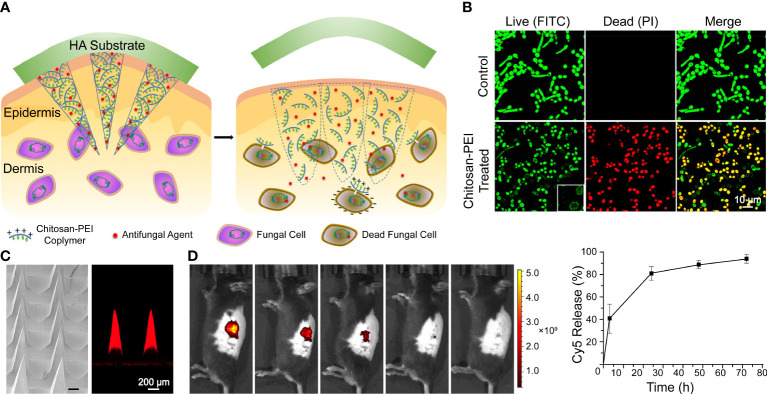
A dissolvable antifungal MN patch with a quick-separable supporting substrate. **(A)** Schematic illustration of the MN patch. **(B)** LIVE/DEAD viability assay of *C. albicans*. **(C)** SEM image of a AmB-loaded MN patch and confocal fluorescence image of Cy5-loaded MNs. **(D)** *In vivo* fluorescence imaging and quantitative release profiles of Cy5 from the MN. Reproduced with permission from Zan et al. (2019).

Engineering methods are used to preserve drugs for intradermal application. Inspired by long-acting implant formulations, Peng et al. (2021a) developed an MN system with AmB-loaded PLGA tips and a quick-dissolving shaft base. The PVP shafts dissolve in interstitial skin fluid and embed the tips intracutaneously for sustained release of AmB. In addition, the base was easily removed and discarded safely in water. A similar concept is also adopted in AmB encapsulation with chitosan-poly(ethylene imine) (PEI) copolymer MNs and an HA supporting substrate, as illustrated in **Figure 2**. Moreover, chitosan-PEI copolymer exhibited fungicidal activity and a synergistic effect with AmB; treated with the copolymer, the surface of fungi became porous and resulted in higher susceptibility to AmB (Zan et al., 2019). Itraconazole nanocrystals delivered in PVP/PVA MNs also exhibited a longer residence time and higher distribution in both concentration and depth compared with conventional creams and needle-free patches in ex vivo porcine skin (Permana et al., 2020).

Furthermore, MN-based intracorneal drug delivery for the treatment of fungal keratitis has shown considerable progress (Roy et al., 2019; Albadr et al., 2022; Suriyaamporn et al., 2022). Adopted from contact lens design features, ocular patches with a curvature that fits the cornea increase the contact area, provide uniform MN insertion, and reduce discomfort in application (Roy et al., 2019). The concerns about the damage of corneal epithelium are greatly minimized since it was reported that the punctures of MN patches on the cornea recovered within 12 hours and could be optimized by polymer formula and MN shapes (Than et al., 2018; Shi et al., 2022a). Similar to intradermal delivery, the drug was not widespread within the eye globe and plasma (Roy et al., 2019). Suriyaamporn et al. (2022) fabricated double-layered MNs with chitosan and PVA for the outer layer and fluconazole microemulsion as stuffing. The reduced lag time and increased permeability indicated a combination mechanism of corneal barrier bypassing and surfactant-based optimization of hydrophobic drugs. However, liposomal AmB delivered by MNs showed lower antifungal activity compared with free AmB MNs (Roy et al., 2019). Further investigations into the underlying mechanism are needed for a comprehensive understanding to the delivery of antifungal drugs.

The aforementioned designs of MNs were generally based on the biodegradability and mechanical properties of polymeric materials. One innovative approach involved is to create a porous MN “cage” made of a combination of biodegradable and non-degradable materials for the purpose of encapsulation of beneficial bacteria, making it an easily removable implant. The immobilized *Bacillus subtilis* was found to be safe for the host’s immune system and host. They secrete a range of potential antifungal agents, such as surfactin. Moreover, both pseudohyphae of C. albicans.and the skin inflammation were minimized (Wang et al., 2020a).

Microneedling therapy is reported to induce epidermal differentiation, growth factor expression, inflammation, dermal remodeling and indirect activation of inflammatory cells. (Zeitter et al., 2014; Schmitt et al., 2018). Although differences exist between procedures of microneedling therapy and MN-based drug delivery, they share similar micro-invasions consequences.

##### 3.1.2.4 Carrier devices

Carrier devices made from polymers are applied topically or inserted into body cavities for sustained drug release. Intravaginal rings are flexible elastomeric toroidal devices that are easily inserted into the vagina by women for hormone replacement therapy or birth control (Roumen, 2008; Tietz and Klein, 2019). In recent studies, intravaginal rings have been developed for microbicide delivery and show potential in treating infectious diseases such as vulvovaginal candidiasis and bacterial vaginosis (Arany et al., 2021; Tiboni et al., 2021). Tiboni et al. (2021) 3D printed a clotrimazole-loaded intravaginal ring made of thermoplastic polyurethane. Clotrimazole was added to castor oil-covered polyurethane pellets and then extruded into filament at 190 °C to homogenize the material. Drug release in vaginal fluid analog was 13.4% in the first week and was maintained at a high concentration above the MIC value of *C. albicans in vivo*. Another manufacturing scheme involved fabricating a hollow drug reservoir, then filling it with drugs, and fixing the drug with excipients. Such devices could be manufactured in extreme conditions, regardless of the drug stability, and refilled if necessary (Arany et al., 2021).

**Figure 3:**
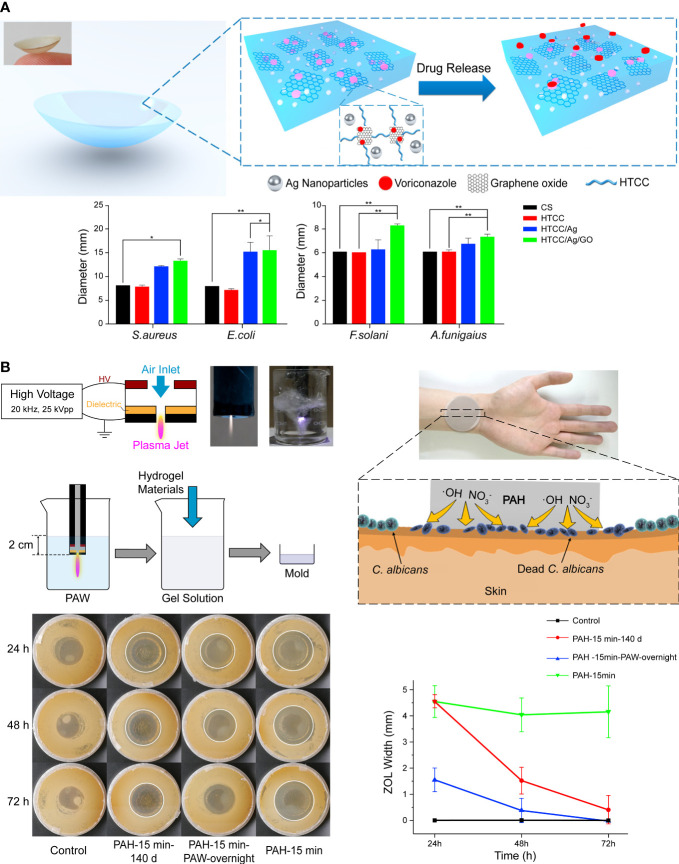
**(A)** Schematic illustration of hydrogel contact lens loaded with conventional drugs. The addition of graphene oxide increased the antifungal activity. Reproduced with permission from Huang et al. (2016). **(B)** Antifungal hydrogel based on the encapsulation of ROS. Reproduced with permission from Liu et al. (2019).

The comfortable structure of contact lenses and advances in the hydrogel are adopted for sustained ocular drug delivery (Ciolino et al., 2011; Phan et al., 2014; Gallagher et al., 2017). Huang et al. developed an electrostatic cross-linking hydrogel-based contact lens by combining materials with innate antimicrobial activity, such as quaternized chitosan, silver NPs, and graphene oxide (**Figure 3A**). The composite was softer and more flexible but with high mechanical properties for contact lens manufacture. Voriconazole was loaded onto the lens by the graphene oxide and released with an initial burst phase followed by a slow, sustained release. *In vivo* results indicated that the AgNPs delayed keratitis (Huang et al., 2016). Liu et al. (2018) demonstrated that AgNPs loaded onto commercial contact lenses with a polydopamine coating for antimicrobial improvement achieved stable visible light transmittance in a simulated clinical application with *A spergillus fumigatus* infected rabbit. However, they turned yellow cultured with high concentrations of bacteria. It indicates that the stability and reliability of the products are significant for the clinical translation. Furthermore, given that wearing contact lenses is a major risk factor for infectious keratitis (Ting et al., 2021), antimicrobial materials are valuable for both therapeutic use and conventional vision correction. However, normal microbiota maintains the ocular health (Wang et al., 2020c), leaving a subtle distinction between appropriate precautions and overuse of microbicide to be studied.

Apart from metallic NPs, essential oils, antimicrobial peptides (AMPs), and other compounds with antifungal activities can also be delivered with polymers, and some exhibit synthetic effects (Yang et al., 2018; Miranda et al., 2019; Olad et al., 2020; Parolin et al., 2021). Reactive oxygen species (ROS) is one of the main effectors of antifungal immunity, but excessive ROS cause tissue injury and inflammation (Branzk et al., 2014). Therefore, capturing and reservation ROS for a controlled release is a promising approach in antifungal therapy. Liu et al. replaced normal water with plasma-activated water rich in free radicals and synthesized a polyacrylamide hydrogel called plasma-activated hydrogels (**Figure 3B**). The polymer network of hydrogel preserved short-lived free radicals such as hydroxyl radicals and nitric oxide for 140 days and exhibited strong antifungal activity, although it faded faster than freshly made hydrogel (Liu et al., 2019).

### 3.2 Systems based on the innate antifungal activity of polymers

Synthetic and modified polymers seem to be further away from clinical translation compared to the delivery systems that are approved antifungal drugs on polymers known to be safe. Despite the biocompatibility concern, polymers with innate antifungal activities remain promising strategies to overcome or even reverse the increasing risk of antifungal resistance primarily based on inherent properties like the surface charge that is hardly adaptive.

Some natural polymers possess antifungal activities. For example, chitosan is a natural cationic polysaccharide derived from chitin and is a structural element in the exoskeleton of crustaceans and fungal cell walls (Hamed et al., 2016). Its fungicidal mechanism includes electrostatic interaction-induced leakage forming a dense film and gene expression alteration (Lopez-Moya et al., 2019; Matica et al., 2019). The positively charged chitosan molecules bind to the phospholipids by electrostatic interaction, stiffen the membrane regions and increase the differences in fluidity between various regions, causing membrane permeabilization (Palma-Guerrero et al., 2010). Although the antifungal activity of chitosan are restricted, and some fungi are resistant to it, they have many chemical groups for functionalization and modification to improve the antifungal effects and expand aditional activities (Palma-Guerrero et al., 2010; Hoque et al., 2016; Fan et al., 2018; Hassan et al., 2018; Mi et al., 2018). For example, multi-aminoethyl and phosphoryl groups can increase the antifungal activity of chitosan and the derivatives have good water solubility and low toxicity to cells (Fan et al., 2018).

HA is a natural, negatively charged glycosaminoglycan polymer that exhibits dose-dependent growth inhibition of *Candida* species and a higher molecular weight leads to stronger activity (Sakai et al., 2007). The sensitivity varies between the strains. For example, growth inhibition of *C. albicans* ATCC 18804 at 1.0 μg/ml (2000 kDa) reaches 40%, whereas the growth of *C. albicans* ATCC 90028 and 90029 is not suppressed at 2 mg/ml (1837 kDa) (Sakai et al., 2007; Ardizzoni et al., 2011). Although the mechanism remained unknown, it was speculated that its massive carbohydrate structures were involved in fungistatic activity (Kang et al., 2011). The antifungal activity of HA was unchanged after conversion into hydrogel (Fahmy et al., 2015). However, in studies on artificial saliva, HA inhibited the candidacidal activities of lysozyme and peroxidase system probably by restricting their diffusion (Kang et al., 2011).

**Figure 4:**
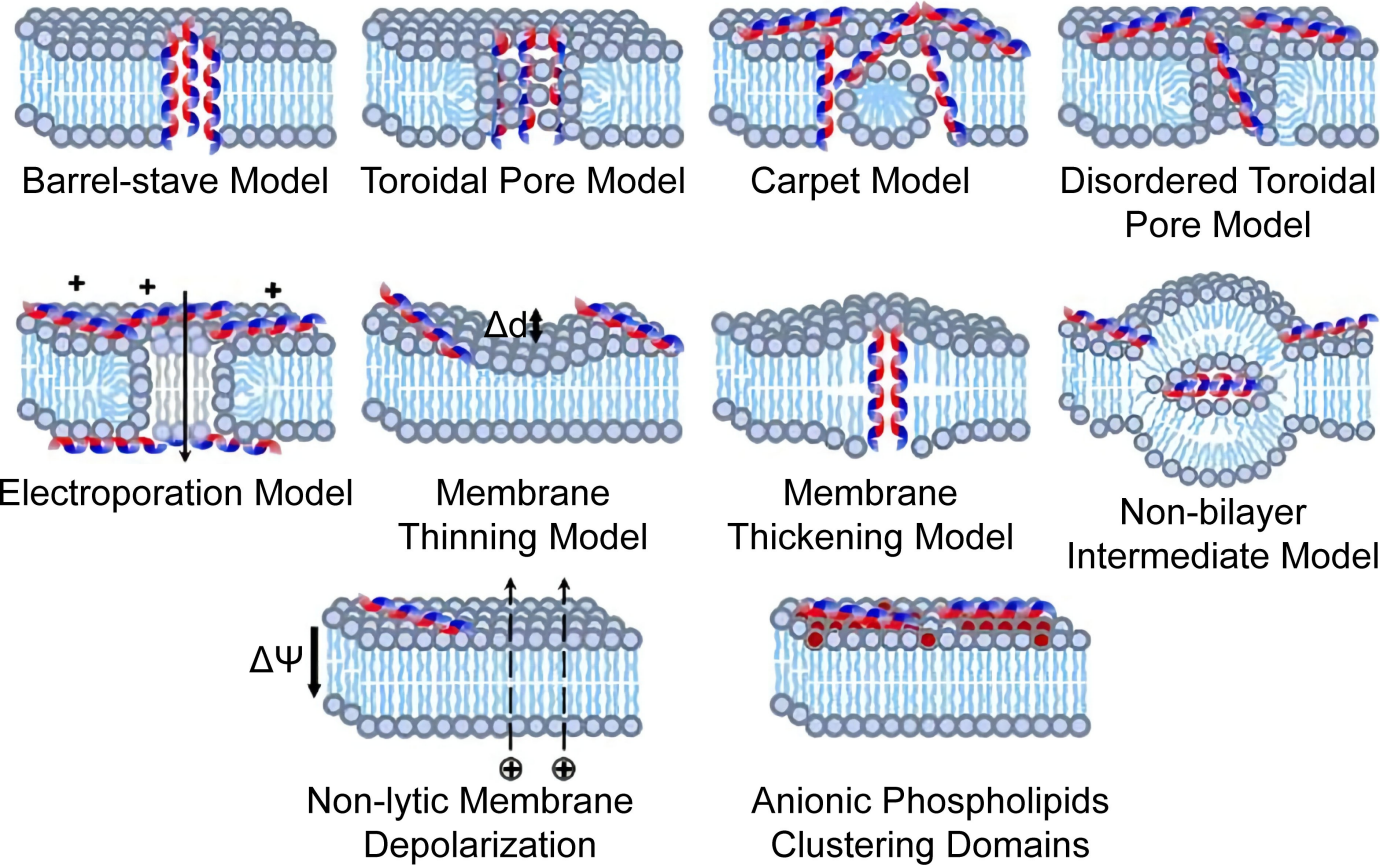
Proposed models of membrane destructive and nondestructive-disturbance antimicrobial mechanisms of AMPs. Reproduced with permission from Yu et al. (2022).

AMPs are abundant and diverse groups of host defense molecules produced by all organisms. Their antimicrobial activity is correlated with their unique pattern of chemical structure that can be imitated in material design. Numerous natural AMPs are recorded and annotated, and the development of synthetic AMPs never ends (Wang et al., 2016; Ramamourthy et al., 2020; Tallet et al., 2022). Their antifungal mechanisms involve pore formation, mannan-binding, nucleic acid, and cell wall inhibition (De Cesare et al., 2020). Their membrane disruption depends on the electrostatic and hydrophobic interaction, as illustrated in **Figure 4**. Moreover, they are simple to imitate with synthetic polymeric materials (Rank et al., 2017; Yu et al., 2022). Schaefer et al. synthesized a series of cationic polyacrylamide terpolymers consisting of different ratios of positively charged, hydrophilic, and hydrophobic monomers and varying degrees of polymerization. It was concluded that shorter polymers with high hydrophobicity tended to be more toxic to *C. albicans*, but hemolytic activities increased with hydrophobicity. Furthermore, the results revealed that cyclic and shorter linear aliphatic functionalities outperform their branched derivatives. The terpolymer with the most potential for therapeutic application exhibited high antifungal activity against drug-resistant fungi and had good biocompatibility that outperformed AmB (Schaefer et al., 2021). Similar results were observed with nylon-3 copolymers which decreased the MIC of azoles and AmB against some selected fungi, highlighting the potential of combination therapy (Rank et al., 2017). A study of aliphatic polyesters found that hydrophobic interaction induced the damage of fungal cell walls and membranes (Cheng et al., 2022). Mohamed et al. (2015) highlighted the antimicrobial activity of cations by demonstrating that a higher degree of quaternization and glutaraldehyde cross-linking of the hydrogel resulted in a strengthened positive charge and more antibacterial activity. The electrostatic interaction also mediates the fungicidal activity of two-dimensional nanomaterials. Saha et al. (2022) wrapped MoSe_2_ nanosheets with chitosan through liquid phase exfoliation and observed distinct damage in the membrane and filament of treated fungi of different genera. The mechanisms were concluded to be electrostatic adhesion, physical piercing, and leakage-initiated death. Inspired by ϵ-polylysine, a cationic antibacterial peptide, poly(DL-diaminopropionic acid) was developed for its substantially increased charge density. The mechanism of action involves electrostatic attaching, both energy-dependent and independent uptake, and ROS-related cell apoptosis and death. These significant fungicidal activities against both planktonic and biofilm forms and their safety *in vivo* suggest that polymers are potentially new antifungal agents (Zhang et al., 2022b).

Hydrophilic polymers exhibit antifungal activities through other mechanisms. For example, a series of cross-linked hydrogels were developed from terpolymers of acrylonitrile, acrylic acid, acrylamide, or its sulfonic acid derivative. Their antimicrobial activity is attributed to cellular stress induced by pH reduction and diffusion of the active monomers (Farag et al., 2020). Stereochemical materials represent a new design concept for fungistatic activities, but it is more suitable for contamination prevention than infection treatment and is thus mostly used in surface modification of medical apparatus and instruments. This will be reviewed in Section 4.2.

The development of novel antifungals is a broad topic. Polymers has attracted special interest owing to the ability to target the inherent properties of fungi, such as the surface charge, and function through physical mechanisms to reduce drug resistance. Correspondingly, their selectivity between microorganisms and host cells is less reliable. Numerous research has focused on topical applications instead of IFD treatment, but the antifungal activities have been tested mainly on *C. albicans.* As previously mentioned, dermatophytes caused the majority of superficial mycosis. Besides, their metabolism and resistance differed greatly from the *Candida* spp. Therefore, the design of antifungal trial needs to be more representative. Moreover, limited *in vivo* toxicity, pharmacodynamics, and pharmacokinetics reports hinder its potential application of polymer-based antifungal in IFD treatment. Besides, a large number of studies remained at the efficacy level, and more studies investigating the underlying mechanism of materials against mycoses are urgently needed.

### 3.3 Photodynamic therapy with polymer-enhanced photosensitizer

**Figure 5:**
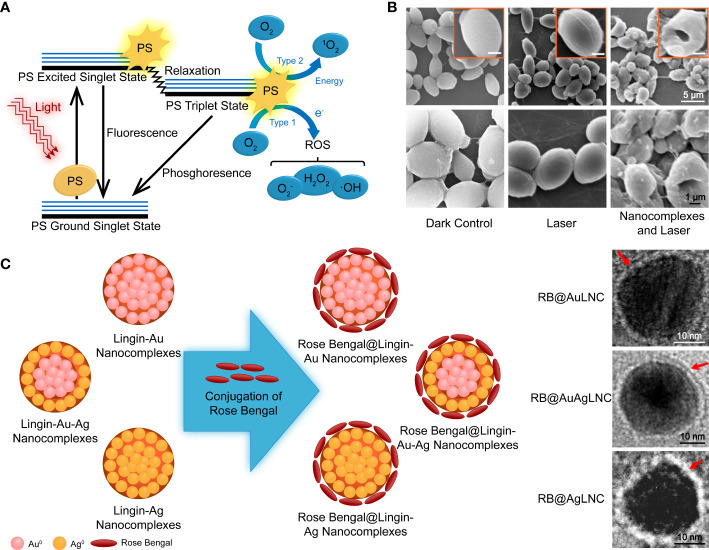
**(A)** Schematic illustration of photodynamic therapy, including the Jablonski diagram. Reproduced with permission from Dai et al. (2012). **(B)** SEM images of PDT-treated and control fungal cells. **(C)** Schematic diagram of the synthesis of PS nanoconjugates by incorporating rose bengal on lignin-metallic and lignin-bimetallic nanocomplexes. Reproduced with permission from Chandna et al. (2020).

Photodynamic therapy is a modern strategy that involves three nontoxic principal components: a photosensitizer (PS), light of an appropriate wavelength, and oxygen molecules. The PSs are usually nontoxic and light-sensitive dyes that can be excited to the first excited singlet state by photons, then relax to the triplet state, interact with molecular oxygen in type I and type II pathways, and generate ROS and singlet oxygen (^1^O_2_), respectively (**Figure 5A**) (Dai et al., 2012). Its antifungal mechanism involves oxidative damage of cell walls and membranes, achieved through the generation of extracellular ROS and photodamage to multiple intracellular targets by oxidizing species that changes the enzyme activities, lipid peroxidation, lyses of cell organelles and membranes, and finally, resulted in cell death (**Figure 5B**) (Gonzales and Maisch, 2012). PDT has been increasingly applied in clinics to treat superficial and localized mycoses, such as onychomycosis and oral candidiasis, due to its multi-factorial and nonspecific antimicrobial properties against antibiotic-resistant species (Shen et al., 2020). However, PDT of cutaneous and subcutaneous fungal infections showed a lower response rate. Therefore, there is increasing interest in strategies to increase the penetration of both PSs and the corresponding light (Shen et al., 2020).

Porphyrin and its derivatives are the major known PSs. However, other designs based on transition metal complexes, aggregation-induced emission luminous, and nanostructures with semiconducting or photoactive materials involved exist (Escudero et al., 2021; Zhou et al., 2021b; Nguyen et al., 2022). Polymer-based strategies are necessary and convenient for biocompatibility promotion, water solubility increase, and aggregation prevention. Furthermore, they are convenient for targeting and sensitizing functionalization (Hutnick et al., 2017; Dos Santos et al., 2021; Zhang et al., 2022c).

Similar to the antifungal drugs, a large number of classic PSs such as phthalocyanines (Pcs) and protoporphyrin (Pp) are hydrophobic, and their aggregation in an aqueous medium significantly reduces the photoactivity (Kwiatkowski et al., 2018). Thus, a polymer-based delivery system is necessary for PSs. Zhang et al. (2022c) combined Pp IX with PEI and further linked the compound with carboxymethyl chitosan (CMC) through EDC/NHS reaction, creating the PS NPs. The CMC-PEI-Pp IX NPs showed remarkable wide-spectrum antimicrobial PDT performance. It had higher ^1^O_2_ generation and microbe entering compared with free Pp IX. Additionally, it had good biocompatibility and stability.

Hsieh et al. (2019) encapsulated iron (III) Pc with chitosan and tripolyphosphate NPs for PS delivery. This increased PDT performance, uptake by fungus, and biocompatibility. Its antifungal activities against adherent *C. tropicalis* were distinctly lower compared with planktonic fungi; although pseudohyphae were reduced, the fungi survived. However, with subsequent treatment with flucytosine, 90% of the adherent fungi were eradicated, indicating antifungal treatments are complementary to PDT. Notably, the differential results from the reversed treatment sequence suggest a complicated synergistic effect to be revealed.

Zinc phenyl-thio-Pc and AmB encapsulated in PLGA nanocapsules is another example of combined therapy. It showed little cytotoxicity at high concentrations of murine fibroblasts without irradiation. The dose-dependent fungistatic effect on *C. albicans* increased with irradiation (Evangelista et al., 2021). Whether a synergy exists between AmB and the phototoxic agent or the phototoxic agent is simply an additive remains to be validated. However, it’s speculated that the membrane permeabilization caused by AmB improves the uptake of nanocapsules as they are negatively charged and electrostatically repulsed to fungi (Evangelista et al., 2021).

As previously mentioned, a micelle is a convenient delivery system for solubility, stability, and permeability enhancement. Loading curcumin, a naturally occurring PS, onto Pluronic^®^ F-127 micelles increased the antimicrobial activities of PDT against pathogens that cause dental caries. However, the system suffered the dilution-disintegration problem (Dos Santos et al., 2021). Hutnick et al. (2017) used poly (amidoamine) dendrimers as a framework for unimolecular micelle vehicles, with the chain end PEGylated for better water solubility and to prevent nonspecific adsorption. Silicon Pc 4 was encapsulated into the hydrophobic core of the dendrimers driven by host-guest supramolecular interactions and showed no reduction in ROS generation. However, the internalization was slightly lower compared with free Pc 4. In addition, their chitin-targeted counterparts functioned by a chitin-binding peptide, was even less effective in the cell uptake trial. Further investigation into the mechanism of such inhibition is needed to improve PS designs and to understand the differences between drug and PS delivery.

The aforementioned examples are based on polymeric vehicle delivery by non-covalent combination with photoactive agents. Covalent strategies are helpful in PS modification and do not compromise the energy conversion performance of PSs. Ruiz-Gonzalez et al. (2017) synthesized cationic dendrimeric Pcs with Zn(OAc)_2_ in the center, and the zinc compounds displayed higher ^1^O_2_ yields in the solution, resulting in a higher microbial reduction upon irradiation. The bulky arms of the dendrimer prevented aggregation, and the large dimension was anticipated to extend circulation. Lignin is an economical, eco-friendly biopolymer and a waste product of the paper pulp and bioethanol industries. It is taken as an example for covalent PS modification rich in phenolic, aliphatic, and carboxylic groups. These provide various properties and reaction sites for chemical reactions (Boerjan et al., 2003). A study described a PS NP system of the photoactive azo dye functionalized lignin incorporated with zinc oxide NPs (Jose et al., 2021). Chandna et al. (2020) created a photodynamic nanoconjugate with two components that have PDT activity, noble metal NPs, and a xanthene dye (**Figure 5C**). Rose bengal was conjugated to the surface of lignin-capped metallic and bimetallic nano complexes *via* ester bonds. In the presence of lignin-capped AgNPs, the PSs exhibited less antifungal activity without irradiation. The PS was further incorporated into transparent and pH-responsive hydrogels for controlled release of the nanoconjugates. However, it was found that this did not increase the antifungal efficiency in a significant way.

Previous studies have attributed the increased cellular uptake and photodynamic inactivation efficacy to the surface charge of the PS system. For example, (Zhou et al., 2021a) demonstrated that the cationic PS selectively lit up the mitochondria of fungi based on the inherent discrepancy in surface charge between fungi and mammalian cells, as well as the negative mitochondrial membrane potential. Another study of NP-encapsulated curcumin supports the importance of electrostatic interaction. In contrast to the enhancement of cationic carriers, anionic NPs lost their antifungal effects due to electrostatic repulsion (Sakima et al., 2018). However, in the study of Ruiz-Gonzalez et al. (2017), cationic dendrimeric octacationic Pcs showed no significant improvement in photoinactivation performance compared with tetracationic analogues, although the cellular uptake has not been well studied. Dibona-Villanueva and Fuentealba (2021) proposed that the promotion of photodynamic inactivation was because of chitosan, which is covalently linked to the photodynamic agent, attached the conjugate to the surface of the pathogen by electrostatic interaction. Therefore, ROS is generated in the vicinity of the cell wall. The CMC-PEI-Pp IX NPs displayed a negative charge as a whole particle, and the researchers attributed the increased NP-access to the electrostatic interaction of microbial cells between some local positive charge of the materials and the membrane of microbes (Zhang et al., 2022c). Tang et al. (2018) found that the conjugates, especially the quaternized conjugates of chitosan oligosaccharides and Pc, achieved higher cell uptake in aqueous media and showed a stronger antifungal effect against *C. albicans*. However, quaternization of CMC conjugated with Pc caused decreased cell uptake and reduced PDT activity (Tang et al., 2020). The phenomenon was thought to be caused by the electrostatic activity from the negatively charged carboxyl group nearby. Hydroxyl radical scavenging activity of quaternized CMC also possibly participated in the function decline (Guo et al., 2008; Tang et al., 2020). The contradictory results of the studies indicate more complicated interactions inside the PS system and between the microbes than just the electrostatic interaction.

Antimicrobial PDT is a localized and topical treatment since the photocytotoxic reactions occur only within the area of PS distribution, and the visible light cannot penetrate deeply into tissues (Kwiatkowski et al., 2018). A potential direction involves the upconversion NPs triggered by deep-penetrated near-infrared rays (NIR), converting NIR into visible emission light to activate the conjugated PSs. (Zhang et al., 2018) synthesized a NIR-triggered PS system with upconversion of NPs, Pc-based PS, and a PVP coating. Few *C. albicans* survived a 20 min excitation of a 980 nm laser *in vitro* at the concentration of 200 μg/mL. However, the killing of microbial cells’ efficiency was reduced as the NIR was still attenuated by the obstructed tissue. This technique could potentially be used for treating subcutaneous lesions, but it cannot reach deeper organs.

Systems based on bioluminescence resonance energy transfer (BERT) represent another potential solution to overcome the limitation of external light sources for the treatment of IFD. Kim et al. (2015) used self-illuminating quantum dots for PDT of cancer. The PS of bioluminescent quantum dots and their activator were given sequentially, and this significantly increased the survival time of tumor-bearing mice. (Yuan et al., 2015) were the first to apply an enzyme cascade-based BERT system for antimicrobial activity. The bio-illuminating element was based on the cascade reaction induced by glucose oxidase and horseradish peroxidase with the addition of glucose and luminol.

With the light penetration problem solved, microbe targeting has become the most promising direction. In contrast with solid tumors, bacteria and fungi scatter between and inside the host cells which requires much higher specificity. Therefore, intracellular targeting and activation could induce more selective photodynamic inactivation and spare more host cells (Zhou et al., 2021a). Protective agents that enhance the resistance of host cells or quench ROS directly are yet to be fully explored (Lobanov and Uzdensky, 2009; Liao et al., 2021). For similar reasons, photothermal therapy is restricted for antimicrobial application. Although the thermotolerance of pathogens is generally better than human somatic cells, fever-range temperatures could improve the immune-protective mechanisms (Evans et al., 2015). Photothermal techniques has also been used synergistically with conventional antifungals to improve drug delivery (Zhang et al., 2020b; Ji et al., 2021).

## 4 Polymer-based strategies in fungal disease prevention

### 4.1 Active and passive immunization

Vaccination is one of the major prevention and control measures for infectious diseases. Currently, there are no licensed fungal vaccines. Various factors, such as molecular complexity of eukaryotic pathogens, reliance on cellular immunity, and characteristics of the target population, have hindered the development of fungal vaccines. Studies have aimed at overcoming the above limitations. A candidate vaccine for recurrent vulvovaginal candidiasis has completed the phase II trial, and has shown great clinical potential (Edwards et al., 2018). Applications of polymeric materials in vaccines is based on two aspects: as adjuvants that facilitate potent immune responses and as carriers that protect antigens and expand the delivery routes.

In vaccine formulations, adjuvants enhance immune responses and increase vaccine efficacy, of which aluminum-containing adjuvants were the first to be approved and are the most widely used due to their good safety. Even though they are effective at enhancing antibody production, their ability to improve cellular immunity is limited (Hogenesch, 2013).

An ionic cross-linked thermosensitive hydrogel composed of chitosan and β-glycerophosphate is used as both the carrier and adjuvant in experimental vaccines with epitope C from heat shock protein 90 against systemic candidiasis. The hydrogel recruits immune cells at the injection site, improves cellular uptake as well as endosomal escape of the antigen due to its buffering capacity, and facilitates the development of long-term humoral immunity through sustained antigen release. The presence of the hydrogel evokes strong Th1, Th2, Th17, and CTL responses with improved cytokine secretion (Li et al., 2021c). The polymer-based NPs are also used for antigen delivery to achieve controlled release. Ribeiro et al. (2013) encapsulated DNA antigens with PLGA NPs, which exhibited a similar level of immune responses to that of naked DNA vaccine when administered *via* the intramuscular route at much lower concentrations. Moreover, PLGA was used to encapsulate cytosolic antigens of *C. albicans* and further entrapped into plasma beads for improved protective immunity. The survival rate was increased by 40% a month after a lethal dose of *C. albicans*, compared to mice immunized with antigens and conventional adjuvant (Ahmad et al., 2015). Adjuvant effects of PLGA are partially mediated by dendritic cells through PLGA-induced maturation and enhanced antigen cross-presentation abilities (Srinivasan and Babensee, 2021).

The mucosal immune system is an important part of the human immune system, and mucous membranes are susceptible sites for opportunistic fungi. Mucosal vaccination induces dual layers of protective immunity at the mucosal surface and in the systemic compartment (Kiyono et al., 2021). Appropriate materials can improve mucosal adhesion, prolong antigen release and enable co-delivery with other active molecules (Amidi et al., 2007; Qin et al., 2020).

Delivery of immune modulators up-regulates the immune system and protects against pathogenic fungi. The P10 peptide, a stimulator of cellular immunity, can enhance the therapeutic efficacy of pharmacotherapy against paracoccidioidomycosis. The encapsulation in PLGA eliminated the requirement for additional adjuvant and reduced the peptide amount by 20-fold (Amaral et al., 2010). The carrier itself may have immunomodulatory activities. Farace et al. (2016) reported that chitosan or poloxamer coated lipid carriers mediated the induction of both innate and adaptive immunity response.

Even though antifungal immunity has long been attributed to innate immunity and the cellular arm of acquired immunity, the potential of protective and therapeutic antibodies against fungal infections have been reported (Pathakumari et al., 2020; Ulrich and Ebel, 2020). Passive immunotherapy with specific humanized monoclonal antibodies is a more substantial therapy, as immunocompromised patients are very highly susceptible to IFD. All commercial antibodies are administered *via* the parenteral route as immunoglobulin molecules can be inactivated by proteases in the digestive tract when orally administered. The pH-sensitive polymer-based carriers are of great potential as oral monoclonal antibody delivery systems. They promote the traversing of antibodies from the intestinal epithelial cells into systemic circulation (Tashima, 2021).

### 4.2 Antifungal medical devices

Bacteria and fungi, *Candida* spp. especially, are highly associated with medical device-related infections. The formation of microbial biofilms is a major challenge as they promote drug resistance when compared to planktonic microbials (Francolini and Donelli, 2010). Interactions between bacteria and fungi may provide extra protection against drugs in polymicrobial biofilms (Francolini and Donelli, 2010). Intravascular catheters are a major risk factor for candidemia, regardless of the origin of pathogens, and should be removed as soon as suspected (Kullberg and Arendrup, 2015). Moreover, additional surgeries are usually required to remove or replace implanted devices (Kojic and Darouiche, 2004). Antifungal materials and coatings have a great potential in medical device-related infection prevention. Strategies against microbial colonization have been previously summarized (Ricardo et al., 2020). Herein, we review several functionalized polymers.

For non-releasing materials, antifouling surfaces are usually realized by increased hydrophilicity or electrostatic repulsion, and contact-killing activities can be mediated by immobilized antimicrobials, AMPs, and quaternary ammonium compounds among others (Ricardo et al., 2020).

An interesting concept involves capturing and quarantining the fungi, which isolates them from the internal environment instead of killing them. Li et al. created a “spore prison” with menthol modified 2,4,6-Trichloro-1,3,5-triazine that firmly confined the fungi in menthoxy units for suppression of spore spread and germination (Li et al., 2021b). The spore prison potentially avoids the development of fungal fragment-induced pro-inflammatory responses (Oya et al., 2019). Even though the killing efficiency of implants is unlikely to induce Jarisch-Herxheimer reactions due to limited contact areas (Muscianese et al., 2020), the superiority of fungicidal materials against fungistatic ones has yet to be determined.

For releasing materials, the main problem is exhaustion of the antimicrobial agent, especially for dentures that are faced with continuous microbial contamination. (Sun et al., 2013) grafted poly(N-vinyl-2-pyrrolidinone) onto surfaces of conventional denture materials for antifungal drug encapsulation to achieve sustained antifungal release without compromising their mechanical properties. The drug release duration lasted from weeks (chlorhexidine digluconate) to months (miconazole). Innovatively, the drug-loaded construction could be quenched by an aqueous solution of the grafted polymer and recharged with different drugs by immersing in the solution. The average longevity for complete dentures is approximately 6 years (Taylor et al., 2021). The ability to reload and replace drugs coordinates antifungal therapies and is applicable for the treatment of other diseases.

Various preventive measures, including personal protective equipment should be used as labor protection appliances or used daily by the susceptible population. Li et al. (2021a) fabricated a nanofibrous membrane loaded with advanced aggregation-induced emission PS that exhibits microbicidal activities under sunlight irradiation. The multi-layered porous structure of the membrane is applicable to masks for interception of pathogenic droplets and aerosols.

Constant fungicidal device use may result in dysbiosis of commensal mycobiota and diseases (Zhang et al., 2020a). Materials with innate fungicidal activities exert stronger effects on bacteria, which exacerbates the imbalance of normal flora and should therefore be regarded as antifungal agents and regulated. The anti-adhesive materials are more suitable for conventional use. Besides, exhaustion and restoration of antifungal activities should be investigated in consideration of service life and use-cost of products.

## 5 Advances in diagnostic and monitoring tools

Host factors, clinical manifestations, and mycological evidence are three main elements of IFD diagnosis, and based on certainty level, they are classified into three levels: “proven,” “probable,” and “possible” (Ascioglu et al., 2002; De Pauw et al., 2008; Donnelly et al., 2020). Confirmed diagnosis of fungal diseases relies on fungal culture and microscopic examination of clinical samples; combined with subsequent antifungal susceptibility tests, it requires a lot of time. Advanced methods, such as PCR-based molecular diagnostics and antigen-antibody reaction-based serological diagnostics, are being developed for more precise species identification in the absence of trained mycologists and rapid detection of antifungal resistance (White and Price, 2021). Non-cultured point-of-care methods have become the trend in diagnosis.

Biosensors are biomacromolecule-integrated devices or probes combined with electronic components for generating measurable signals. They are capable for detection and measurement of very low concentrations of specific pathogens or chemicals (Naresh and Lee, 2021). Pla et al. (2021) designed a biosensor based on oligonucleotide-capped nano-porous anodic alumina for effective detection of *C. auris* that allows accurate detection in an hour without previous sample treatment or amplification steps. The method was based on DNA hybridization and release of entrapped fluorophore. (Bhatnagar et al., 2018) designed a sensitive electrochemical nano-biosensor for diagnosis of invasive aspergillosis *via* detection of the virulent glip target gene. Analytical parameters were determined by probe-target hybridization and intercalation of toluidine blue, and reported in less than 20 min. Components of the fungal cell wall were also distinctive. Concanavalin A and wheat germ agglutinin are lectins that effectively bind glycan and mannan units in yeast cell walls. A novel electrochemical biosensor platform was based on such recognition elements for identification of pathogenic *Candida* species. Evaluation was based on changes in charge transfer resistance, which was affected by variations in dispersion and blocking of sensor surfaces depending on constitutions of different *Candida* cell walls (Sa et al., 2020). Combined methods for diagnosis of fungal species continue to appear. Hu et al. reported a rapid detection method that is based on surface-enhanced Raman scattering with Fe_3_O_4_@PEI magnetic NPs to capture *Candida via* electrostatic interactions and positively charged AgNPs as substrates. Three *Candida* species were tested and clearly distinguished by the advanced analysis method (Hu et al., 2021).

Evaluation of drug sensitivity is important but time-consuming and requires specialized laboratories. Yu et al. (2020) developed a lab-on-a-chip platform for portable detection of genetic markers that are associated with azole resistance and an assorted smartphone system for monitoring and cloud processes. The platform will improve clinical decision making, infection control, and epidemiological surveillance. Another device for antifungal susceptibility determination is based on screen-printed carbon electrodes for real-time monitoring of treated *Candida* biofilm growth. Even though the throughput of the electrochemical method is not satisfying, low costs and the propensity for miniaturization and automation make it a potential alternative for rapid assessment of antifungal activities (Olaifa et al., 2021).

**Figure 6:**
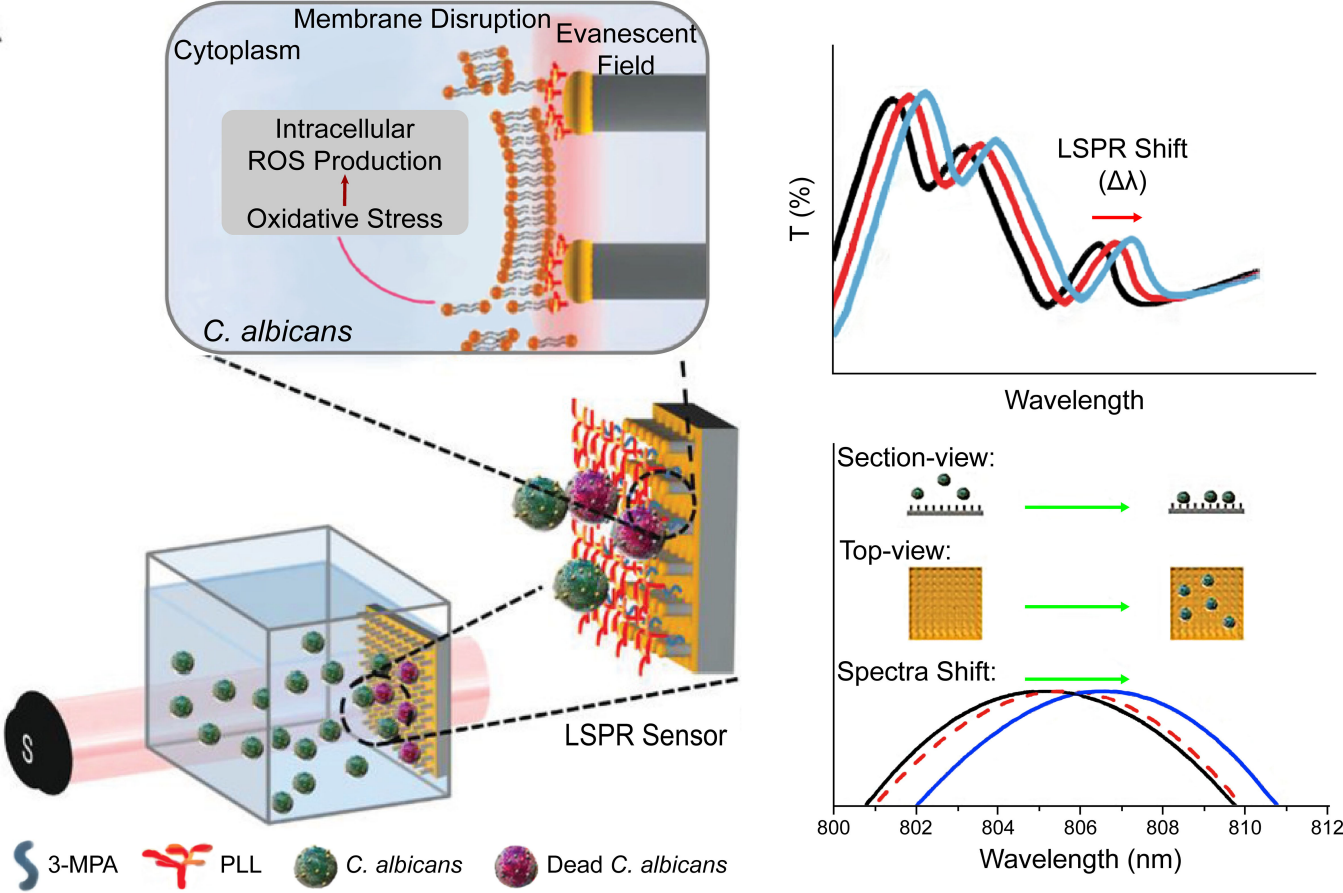
Schematic illustration of the structure and mechanism of a fungicidal sensing platform. The biosensor was based on the shift of localized surface plasmon resonance peaks that resulted from the adhesion of fungal cells to gold nano arrays. The surface modification by the polycationic polymer, poly-L-lysine, increased the attachment of *C. albicans* and provided antifungal properties. Reproduced with permission from Zhou et al. (2022).

“Sense-and-treat” is the cutting-edge concept in health management. Zhou et al. (2022) developed a multi-functional sensing platform that is based on localized surface plasmon resonance sensors and gold nano-match head arrays to monitor the *C. albicans* adhesion (**Figure 6**). Changes in optical performance near sensor surfaces were captured to reflect microbial adhesion, which resulted in shifts of localized surface plasmon resonance peaks. The sensor exhibited higher stabilities and sensitivities when its surface was modified with poly-L-lysine. The polycationic polymer also exhibited fungicidal activities. Even though the readily available samples such as sputum and vaginal fluid are not reliable for distinguishing colonization from invasion, the results are still valuable for IFD diagnosis and self-monitoring of susceptible populations. A detecting platform or method may differently interact with different biomarkers, including proteins and nucleic acids. Studies should aim at improving the sensitivity, specificity and applicability of the platform. Modular designs with variable markers or reagents are potential integrated diagnostic tools for strain identification and sensitivity tests. In pursuit of self-service, studies are aimed at developing portable, point-of-care, and high-sensitive diagnostic and monitoring devices for infectious diseases (Heikenfeld et al., 2019). Futuristic miniaturized hand-held devices for point-of-care detection of fungal diseases should be developed.

## 6 Conclusions and future directions

Polymers are potential fundamental materials for protecting, delivering or integration of active components. They may also be the bearers for functions in drug-free systems. In fungal disease management, innovative solutions for prevention, diagnosis, and treatment have been proposed. Polymeric delivery systems can be highly customized based on chemical structures and properties of loaded antifungals to limit their biotoxic effects and exert synergetic effects. However, only *in vitro* studies are not enough as findings from *in vivo* studies may differ due to the complex internal environment. For example, clearance of NPs by the reticuloendothelial system may result in unwanted drug distribution and impair the original purpose of sustained drug release (Hu et al., 2018). Therefore, there is a need to investigate the *in vivo* pharmacokinetics of delivery systems, particularly those with novel surface modifications. The choices of research model should also be made wisely according to the clinical aims. Studies should focus on systems that are aimed at targeted delivery, including blood-brain barrier permeation for central nervous system IFD treatment and pulmonary concentration against fungal pneumonia. In terms of topical delivery systems, most of the studies have been aimed at improving efficacy and patient experience of pre-existing dosage forms, such as gels and nail lacquers. Target sites have included body skin, nails, oral and vaginal mucous membranes. However, to the best of our knowledge, few studies have specifically focused on treatment of tinea capitis, which usually affects children. Inclusion of aesthetic requirements reflects the compassionate care of modern medicine. Symptomatic relief and masking of lesions should be considered in future studies to improve the physical and mental health of patients.

Drug-free therapies are novel medical applications in polymer science. They are promising solutions to the growing problem of drug-resistant fungi. Previous studies mostly focused on topical applications of such systems. However, unlike aggregated cancer cells, pathogenic fungi spread throughout affected sites, sometimes even in whole organs. The rationality of current systems was based on the fact that the skin and mucous membranes are natural barrier tissues with strong regenerative abilities and thus more tolerant to the microenvironment change. However, cells of inner organs are more sensitive and fragile. Potential systemic applications should aim at improving selectivity at the cellular level to reduce damage to body cells. Long-term observation is also necessary in case some underlying minor irritation is carcinogenic.

With the increasing public health threat of fungal resistance, the “precision medicine” concept has been introduced. Futuristic prevention and diagnostic devices are current hot spots. The design for superficial and deep applications should coincide with the tissue characterization and category of mycoses. The genetic background and underlying diseases target population should also be considered. For superficial mycoses, comfortable protective equipment, handy diagnosis, and provision of health advice are important. Moreover, the combination of smart phones and big data may assist in self-help prevention and treatment of superficial mycosis and reduce public health expenditures. Regarding IFD, timely monitoring of drug susceptibility, pathogen load, and reaction to treatment is the future direction. Point-of-care molecular diagnosis may accelerate the clinical translation of novel RNA-based therapies (Bruch et al., 2022). In conclusion, polymer-based strategies have promising applications in prevention, diagnosis, and treatment of fungal infections and may achieve closed-loop management in future.

## Author contributions

Writing - original draft, SW; writing - review and editing, WG; data curation, HZ and HM; resources, RyL and DG; supervision, XZ; funding acquisition, RL; conceptualization, WQ; manuscript revision, BL and JS. All authors contributed to the article and approved the submitted version.
